# Autapses promote synchronization in neuronal networks

**DOI:** 10.1038/s41598-017-19028-9

**Published:** 2018-01-12

**Authors:** Huawei Fan, Yafeng Wang, Hengtong Wang, Ying-Cheng Lai, Xingang Wang

**Affiliations:** 1 School of Physics and Information Technology, Shaanxi Normal University, Xi’an, 710062 China; 2 School of Electrical, Computer, and Energy Engineering, Arizona State University, Tempe, Arizona 85287 USA

## Abstract

Neurological disorders such as epileptic seizures are believed to be caused by neuronal synchrony. However, to ascertain the causal role of neuronal synchronization in such diseases through the traditional approach of electrophysiological data analysis remains a controversial, challenging, and outstanding problem. We offer an alternative principle to assess the physiological role of neuronal synchrony based on identifying structural anomalies in the underlying network and studying their impacts on the collective dynamics. In particular, we focus on autapses - time delayed self-feedback links that exist on a small fraction of neurons in the network, and investigate their impacts on network synchronization through a detailed stability analysis. Our main finding is that the proper placement of a small number of autapses in the network can promote synchronization significantly, providing the computational and theoretical bases for hypothesizing a high degree of synchrony in real neuronal networks with autapses. Our result that autapses, the shortest possible links in any network, can effectively modulate the collective dynamics provides also a viable strategy for optimal control of complex network dynamics at minimal cost.

## Introduction

An autapse is referred to as a special synapse that connects the axon and dendrites of the same neuron. Autapses were first discovered in 1972 in the pyramidal cell of neocortex cerebri (Golgi preparations of rabbit occipital cortex)^1^. Because of their aberrant and incestuous structure, autapses were originally regarded as “a wiring error” in the development of the brain and therefore were conceived as playing no actual role in the biological functions of the underlying neural network. Twenty-five years later, autapses were found commonly in the neural circuits of the cat’s visual cortex^2^, suggesting that autapses may actually have certain biological usage^3,4^. Sign of ubiquity of autapses emerged subsequently, when they were discovered in multiple areas in the brain such as neocortex, hippocampus, cerebellum, substantia nigra, and striatum^5–8^. There was experimental evidence that autapses are sparse and are present only on a small fraction of the neurons in the underlying brain neuronal network^5,6^. With respect to the functional role of autapses, significant improvement in the spike-time precision of the neocortical interneurons was found in the presence of inhibitory autapses^6^, and excitatory autapses were found to be essential to persistent activities of certain neurons of *Aplysa*^8^. In theoretical and computational studies, issues that have been addressed concerning the roles of autapses include: persistence in recurrent neural networks^9^, generation of oscillatory behavior of a single neuron^10^, switching among distinct dynamical states (e.g., quiescent, periodic or chaotic)^11^, induction of wave patterns in a regular network of neurons^12^, signal detection in stochastic neurons^13^, emergence of coherence resonance in single neurons and in neural networks^14^, and promotion of rhythmic propagation in neuronal networks^15^.

A fundamental issue in complex networks with significant implications to biology and physiology is synchronization^16^, a ubiquitous phenomenon in natural systems^17^. A widely known example is epilepsy, a disorder characterized by seizures, which affects over 50 million people worldwide. The classical understanding is that seizures are associated with hypersynchrony^18^. This principle, however, has been challenged^19–22^, and the dynamical origin of epilepsy in relation to synchrony has been continuously debated^23,24^. As a matter of fact, the lingering and unanswered question in this field of medicine is whether epileptic seizures are associated with an increased or a decreased level of neuronal synchrony. To certain extent, this question may be addressed through the approach of data analysis: by analyzing EEG (electroencephalogram) or ECoG (electrocorticogram) data recorded from epileptic patients and calculating measures of synchrony, one hopes to be able to assess the possible causal role of synchronization in triggering a seizure. Indeed, there were previous efforts in developing seizure detection and characterization frameworks based on partial synchrony among multichannel brain data, with the finding that, depending on the type of seizures, there can be either an enhancement or a reduction in the degree of synchrony^25–27^. The data based approach has one deficiency: it reveals no information about the interplay between seizure and synchrony at the localized neuronal network level, as the EEG or ECoG data reflect only the collective neuronal activities at much larger scales. This leads to a related question: suppose epileptic seizures are indeed associated with synchronization (either enhanced or reduced) in small scale neuronal networks, what characteristic structural features do the networks possess to promote or suppress synchrony? Identification of unusual and unconventional features in the seizure network structure which predominately affect synchronization can potentially lead to a deep understanding of the interplay between seizure and synchrony.

In this paper, we develop the computational and theoretical foundation for hypothesizing that autapses associated with single neurons are capable of significantly modulating synchrony at the network level. In particular, implementing a widely studied nonlinear neuron model on complex networks of different topologies, we assume the existence of autapses on a small fraction of the neurons and investigate quantitatively how global synchronization of the network is affected by the locations, strength, and time delays of the autapses. We find that, in general, the presence of a sparse set of autapses can increase dramatically the odds for the whole network to achieve global synchronization. We develop a criterion that allows us to determine, for a given set of autapses, their optimal locations in the network to maximize synchrony. The implication is that autapses can serve as an effective structural indicator at the single neuron level for anticipating synchronization in networks that contain such neurons. In epilepsy, for example, there are distinct types of seizures that are associated with neuronal networks in different regions of the brain. If it is possible to examine the structural details of the representative neurons in such a network, the presence of autapses would imply a higher probability for synchronization and, consequently, a more appreciable likelihood that synchrony is the culprit of the corresponding seizure. This may provide insights into a possible resolution of the interplay and causal relation between synchrony and certain types of seizures. With the advance of biotechnologies, we hope that it would be possible to test our hypothesis that autapses are correlated with synchrony through structural examination at the single neuron level and dynamics characterization at the network level. In a broader perspective, our finding that autapses, the shortest possible links in any network, are able to modulate the collective dynamics might provide an alternative approach to controlling the dynamics of complex networks, especially for situations where long-distance links are costly and infeasible.

## Results

### Understanding the role of autapse in promoting neuronal synchronization through a toy network model

**Figure 1:**
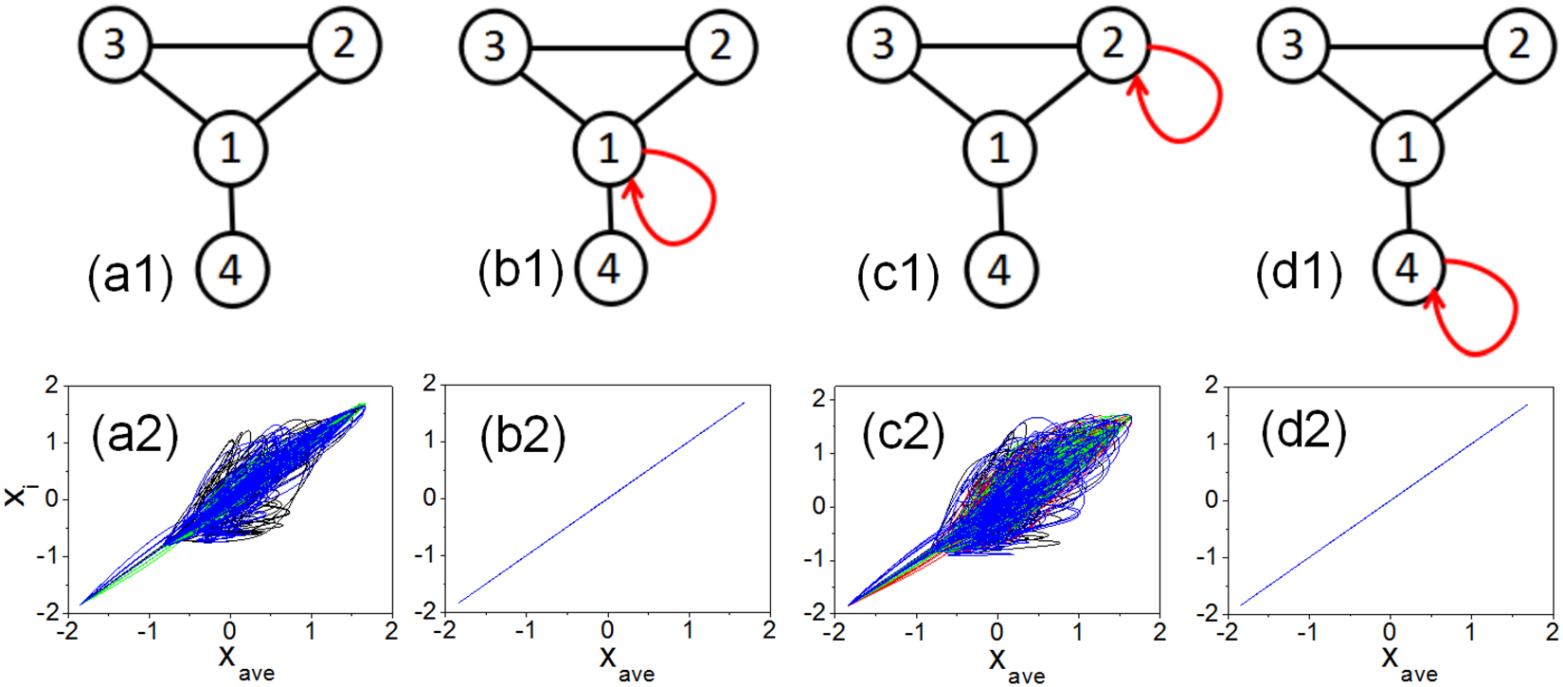
The impact of a single autapse on synchronization in a toy neuronal network. (**a1**) Without any autapse, the network has four nodes and four edges, where each node is a Hindmarsh-Rose neuron. (**b1**–**d1**) Network structure when a single autapse (represented by the red curve with an arrow) is present at node 1, 2, and 4, respectively. (**a2**–**d2**) For the network structures in (**a1**–**d1**), respectively, the dynamical behaviors of the network in terms of synchronization. Shown in each panel is a plot of the *x* variable from each node versus the averaged value of this variable over all the nodes during the time evolution. When there is global synchronization, all the variables are equal to their average value at any instant of time, tracing out a straight line segment along the diagonal. Any deviation from the diagonal signifies lack of synchronization. The uniform coupling parameter is *ε* = 1 and the time delay associated with the autapse is *τ* = 4.

To demonstrate that autapses can promote global synchronization in neuronal networks, we start with a toy model consisting of four coupled neurons (Fig. 1). Mathematically, an autapse can be modeled as a self-loop in the network with a time delay *τ* (see Methods). As shown in Fig. 1(a1), in the absence of any autapse, the network has four nodes and four mutual links (edges). The coupling strength associated with each edge is determined by the parameter *ε* normalized by the nodal degree (including the autapse) (see Methods). We assume that each node corresponds to an idealized neuron, whose dynamics are identical and described by the Hindmarsh-Rose (HR) oscillator^28–32^:

$$\begin{array}{ccc}\dot{x} & = & y+\varphi (x)-z+I,\\ \dot{y} & = & \psi (x)-y,\\ \dot{z} & = & r[s(x-{x}_{R})-z],\end{array}$$

with

$$\begin{array}{ccc}\varphi (x) & = & -a{x}^{3}+b{x}^{2},\\ \psi (y) & = & c-d{x}^{2},\end{array}$$

where *x* is the membrane potential, *y* and *z* represent the transport rates of the fast and slow ion channels corresponding to the spiking and bursting variable, respectively, *I* is the external stimulating current that determines the nature of the isolated neuronal dynamics, e.g., periodic or chaotic. To appreciate the ability of autapses to promote synchronization for complicated dynamics, we choose the parameters of the HR neuron so that it exhibits a chaotic attractor (see Methods).

A single autapse can be present at any node, and there are three distinct cases, as shown in Fig. 1(b1–d1), respectively (due to the symmetry in the network structure, an autapse on node 3 is equivalent to one on node 2). To determine if there is global synchronization in the network, we plot one dynamical variable, e.g. *x*_*i*_(*t*), from each node (for *i* = 1, …, 4), versus the network averaged variable defined as *x*_*ave*_(*t*) = ∑_*i*_*x*_*i*_(*t*)/4 for a relatively large time interval. When the network is completely synchronized, we have *x*_*i*_(*t*) = *x*_*ave*_(*t*) for *i* = 1, …, 4, so the dynamical trajectory will trace out a straight line segment along the diagonal in the (*x*_*i*_, *x*_*ave*_) plane. Any deviation from the diagonal indicates lack of synchronization in the system. From Fig. 1(a2–d2), we see that, without any autapse (a2), the network is not synchronized. When an autapse is present at node 1 or 4, which has the largest and the smallest degree, respectively, there is global synchrony in the network, as shown in Fig. 1(b2) and (d2), respectively. Adding an autapse at node 2 (or 3) will not result in global synchronization (c2). These results indicate that the presence of a single autapse at a proper location in the network can promote synchronization.

Intuitively, from the network synchronization theory^33–47^, the introduction of an autapse is likely to discourage global synchronization because the time delay associated with the autapse effectively makes the dynamics of the neuron hosting the self-loop non-identical to other neurons in the network at which no autapses are present. Previous simulation of an isolated neuron showed^11^ that, with the addition of an autapse, the neuronal dynamics could be changed drastically, e.g., from chaotic to periodic. If the dynamics of the individual nodes are characteristically different, the synchronization manifold existing for identical nodal dynamics may be destroyed^48^. The phenomenon of autapse promoted synchronization, as exemplified in Fig. 1(b2) and (d2), thus seems to be counterintuitive. However, an examination of the dynamical behavior of neuron 1 (or neuron 4) reveals that, even in the presence of an autapse, its trajectory is identical to that without the autapse. This observation suggests that, in spite of the autapse, the network still possesses a global synchronization manifold. In Methods, we provide a heuristic argument for the existence of a synchronization manifold in the presence of an autapse. (It is worth noting that, depending on the time-delay parameter, the dynamics within the synchronization manifold may differ from those of the individual, isolated neurons. For the time-delay parameter used in Fig. 1(b), there are periodic dynamics in the synchronization manifold).

To demonstrate that autapses can promote synchronization in a more quantitative manner, we exploit the concept of master stability function (MSF)^49^. Given a networked system possesses the synchronization manifold, the MSF method proposed in 1998 by Pecora and Carroll^49^ has become the golden standard in the study of synchronization in complex dynamical systems. To appreciate the physical significance of the MSF method, we note that, as the network approaches a global synchronization state, the variables of the nodes approach each other asymptotically due to the mutual entrainment caused by the coupling. The subspace in which the synchronous solution lies is the synchronization manifold whose dimension is typically much smaller than that of the full phase space. Specifically, let ***x***_***s***_(*t*) be the synchronous state of the network and {*δ****x***_*i*_(*t*)} be the infinitesimal perturbations added on node *i* at time *t*. Whether the perturbed system is restorable to the synchronous state can be inferred from the MSF^49^.

$$\delta \dot{{\boldsymbol{y}}}=[{\boldsymbol{DF}}({{\boldsymbol{x}}}_{s})+\sigma {\boldsymbol{DH}}({{\boldsymbol{x}}}_{s})]\cdot \delta {\boldsymbol{y}},$$

with *δ****y*** and *σ* = *ελ* being the perturbation in the eigenmode space and the generic coupling strength, respectively. The quantity *ε* represents the uniform coupling strength, and {*λ*} are the eigenvalue spectrum of the Laplacian matrix defining the network structure (see Methods). Denoting Λ as the largest Lyapunov exponent calculated from the above equation, we have that the variation of Λ with *σ* gives the shape of the MSF curve. When the coupling configuration and the synchronous dynamics (i.e., the dynamics of an isolated node) are given, the MSF curve is independent of the particular network structure. If the dynamics in the synchronization manifold is stable with respect to perturbations in the transverse subspace, synchronization can be physically realized. In this case, the value of Λ is negative for all the transverse perturbation modes *σ*_*i*_ = *ελ*_*i*_ for *i* = 2, …, *N* (*N* is the number of coupled oscillators and the mode associated with *λ*_1_ = 1 describes the motion along the synchronization manifold. See Methods for details). For the general nodal dynamics and coupling configuration, the value of Λ is negative only in certain interval^36,49,50^ of the generic parameter. As such, for a network of coupled nonlinear oscillators to be synchronizable, the necessary condition is that all the generalized coupling strengths of the transverse modes {*σ*_*i*_} fall entirely the interval of negative Λ. (A mathematical formulation of the MSF is given in Methods).

**Figure 2:**
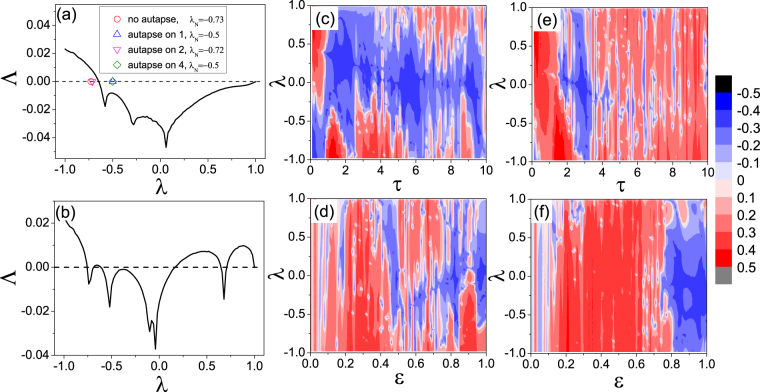
Master stability functions (MSFs) for the network of four neurons. For the toy neuronal network model in Fig. 1, the stability of the MSF, as characterized by Λ, for different combinations of the system parameters. The synchronization state is stable when Λ < 0 for all the transverse modes, and will be unstable when Λ < 0 for any of the transverse modes. All results are obtained by solving Eq. (9) numerically (see Methods). (**a**) For *ε* = 1 and *τ* = 4, Λ versus *λ*. We see that Λ is negative in the region *λ* ∈ (−0.7, 1), indicating that stable synchronization will be achieved when all the transverse modes are located in this parameter interval. (**b**) For *ε* = 0.8 and *τ* = 4, Λ versus *λ*, where now Λ is negative in three subregions: *λ* ∈ (−0.82, −0.75), *λ* ∈ (−0.72, 0.36), and *λ* ∈ (0.61, 0.67). (**c**) For fixed *ε* = 1, contour plots of Λ in the parameter plane (*λ*, *τ*), where the blue color indicates parameter regions of stable synchronization (Λ < 0) and the red color specifies the regions where synchronization is unstable or cannot be realized physically (Λ > 0). (**d**–**f**) Contour plot of Λ in the parameter planes (*λ*, *ε*), (*λ*, *τ*), and (*λ*, *ε*) for fixed *τ* = 4, *ε* = 0.8, and *τ* = 6, respectively. The general phenomenon is that, through variations of the parameters (*λ*, *τ*, and *ε*), the region of stable synchronization can be readily modified.

We calculate the MSF curve for the small neuronal network in Fig. 1 without or with an autapse at each node, to assess the impact of autapse on synchronization. For a fixed value of *ε*, the argument of *σ* (i.e., the generalized coupling parameter) is directly proportional to the eigenvalue *λ* of the network coupling matrix. For convenience, we choose *ε* = 1 and plot Λ versus *λ* (see Methods). Figure 2(a) shows such a plot for *τ* = 4 (the same parameter values as in Fig. 1). We have Λ < 0 for *λ* ∈ (*σ*_1_, *σ*_2_) with *σ*_1_ ≈ −0.7 and *σ*_2_ ≈ 1. The structure of the small neuronal network gives *λ*_1_ = 1 = *σ*_2_ and *λ*_1_ > *λ*_2_ ≥ *λ*_3_ ≥ *λ*_4_. Stable synchronization of the network is thus determined solely by the the smallest eigenvalue *λ*_*N*_: synchronization is (is not) achievable for *λ*_*N*_ > *σ*_1_ (*λ*_*N*_ < *σ*_1_). Without any autapse Fig. 1(a1), we have *λ*_4_ = −0.73. Since *λ*_4_ < *σ*_1_, the network is not synchronizable - in agreement with the numerical result in Fig. 1(a2). When an autapse is present at neuron 1 Fig. 1(b1), we have *λ*_4_ = −0.5 > *σ*_1_, so the network is synchronizable, as indicated by Fig. 1(b2). Similarly, for an autapse at neuron 4 Fig. 1(d1), we have *λ*_4_ = −0.5 > *σ*_1_ so there is synchronization Fig. 1(d2). However, when the single autapse is attached to neuron 2 Fig. 1(c1) we have *λ*_4_ = −0.72 < *σ*_1_, so the network is non-synchronizable, as indicated by Fig. 1(c2).

In general, when the network structure is given so that all eigenvalues {*λ*_*i*_} (*i* = 1, …, *N*) are known, whether stable synchronization can arise is determined by the MSF curve, where the range of the negative values of the MSF, namely the stable region, determines whether all the transverse eigenmodes are stable. For network systems without time delay (the general model studied in literature), the existence and the size of the stable region depend only on the local nodal dynamics and the coupling function, but not on the coupling strength^49,50^. For our neuronal network with autapses, however, the stable region depends also on the coupling strength parameter and the time delay. To uncover the effect of these two parameters on the stable region of the MSF curve, we decrease the coupling strength to *ε* = 0.8 and calculate the corresponding MSF, as shown in Fig. 2(b). Comparing Fig. 2(a) and (b), we see that not only the range but also the shape of the stable synchronization region are modified: the stable region now consists of three separated subregions: (−0.82, −0.75), (−0.72, 0.36), and (0.61, 0.67). To obtain a full picture of the distribution of the stable regions in the three-dimensional parameter space (*ε*, *λ*, *τ*), we show in Fig. 2(c–f) the value of Λ in four different parameter planes. In general, network synchrony is sensitive to the network structure (characterized by the eigenvalue spectrum {*λ*_*i*_}), the coupling parameter (*ε*), and the time delay parameter *τ* associated with the autapse. The sensitive dependence of stable synchronization region on variations in *ε* and *τ* is a typical feature of ragged synchronization^51^. The remarkable phenomenon is that, regardless of the changes in the parameters, insofar as there is an autapse, stable synchronization regions [blue regions in Fig. 2(c–f) persist. That is, for an unsynchronized neuronal networked system, the presence of a single autapse is able to induce stable synchronization.

### Autapse centralities and synchronization optimization

Suppose a small set of autapses are distributed into a neuronal network, to which set of neurons should the autapses be attached so that the network achieves the maximum synchronizability? To address this question for the general case of multiple autapses is difficult. We thus first consider the relatively simple case of a single autapse and develop a theoretical criterion to determine the optimal location for the autapse. Equivalently, it is necessary to find the autapse centrality, a quantity that measures the impact of a specific autapse on the network synchronizability. From the MSF formalism, we have that the autapse induces a shift in the eigenvalues of the network coupling matrix. Specifically, let ***G***^0^ and ***G*** be the network coupling matrices without and with the autapse, respectively. Letting ${\lambda }_{j}^{0}$ and *λ*_*j*_ be the *j*th eigenvalue of the respective matrices, we write the eigenvalue shift as ${\rm{\Delta }}{\lambda }_{j}\equiv {\lambda }_{j}-{\lambda }_{j}^{\mathrm{(0)}}$. In the typical case where the MSF curve possesses only one bounded stable region, the network can reach a global synchronous state only if the eigenmodes in the transverse subspace associated with the largest and the smallest nontrivial eigenvalues (*λ*_2_ and *λ*_*N*_, respectively) are both stable. The impact of the autapse on the network synchronizability can then be characterized by the shifts in the two nontrivial eigenvalues: ${\rm{\Delta }}{\lambda }_{2}^{i}={\lambda }_{2}-{\lambda }_{2}^{\mathrm{(0)}}$ and ${\rm{\Delta }}{\lambda }_{N}^{i}={\lambda }_{N}-{\lambda }_{N}^{\mathrm{(0)}}$, with *i* denoting the neuron that receives the autapse. A large value of ${\rm{\Delta }}{\lambda }_{2}^{i}$ or ${\rm{\Delta }}{\lambda }_{N}^{i}$ signifies a more significant impact. We thus propose the quantities ${\rm{\Delta }}{\lambda }_{2}^{i}$ and ${\rm{\Delta }}{\lambda }_{N}^{i}$ as two autapse centrality measures.

To characterize the autapse centralities quantitatively, we develop a perturbation-based approach by treating the autapse as a small alteration to the network structure. With the perturbation, the normalized network matrix can be written as ***G*** = ***G***^(0)^ + ***G***^(1)^, where ***G***^(1)^ is the perturbation matrix induced by the autapse. Assuming that the autapse is attached to neuron *i* which has degree *k*_*i*_, we have the elements of ***G***^(1)^ as ${g}_{ii}^{\mathrm{(1)}}=\mathrm{1/(}{k}_{i}+\mathrm{1)}$, ${g}_{ij}^{\mathrm{(1)}}=-\mathrm{1/[}{k}_{i}({k}_{i}+\mathrm{1)]}$ if there is a link between neurons *i* and *j*, and ${g}_{ij}^{\mathrm{(1)}}=0$ for other elements. Letting ***e***_2_ = [*e*_2,1_, *e*_2,2_, …, *e*_2,*N*_]^*T*^ be the normalized eigenvector associated with the eigenvalue ${\lambda }_{2}^{\mathrm{(0)}}$ of ***G***^(0)^ [i.e., ${{\boldsymbol{G}}}^{\mathrm{(0)}}{{\boldsymbol{e}}}_{2}={\lambda }_{2}^{\mathrm{(0)}}{{\boldsymbol{e}}}_{2}$], and ${{\boldsymbol{e}}}_{2}^{-1}=[{e^{\prime} }_{\mathrm{2,1}},{e^{\prime} }_{\mathrm{2,2}},\ldots ,{e^{\prime} }_{\mathrm{2,}N}]$ be the left eigenvector of ***G***^(0)^ (i.e., ${{\boldsymbol{e}}}_{2}^{-1}\cdot {{\boldsymbol{G}}}^{\mathrm{(0)}}={\lambda }_{2}^{\mathrm{(0)}}{{\boldsymbol{e}}}_{2}^{-1}$ and ${{\boldsymbol{e}}}_{2}^{-1}\cdot {{\boldsymbol{e}}}_{2}\mathrm{=1}$)^52^, we have

1 $${\rm{\Delta }}{\lambda }_{2}\approx {{\boldsymbol{e}}}_{2}^{-1}{{\boldsymbol{G}}}^{\mathrm{(1)}}{{\boldsymbol{e}}}_{2}=\sum _{i,j\mathrm{=1}}^{N}{e^{\prime} }_{\mathrm{2,}i}{e}_{\mathrm{2,}j}{g}_{ij}^{\mathrm{(1)}}\mathrm{.}$$

For a densely connected network, we have ${k}_{i}\gg 1$ and so ${g}_{ij}^{\mathrm{(1)}}\ll {g}_{ii}^{\mathrm{(1)}}$. Under the approximation ${g}_{ij}^{\mathrm{(1)}}=0$, we have that the matrix ***G***^(1)^ has only one non-zero element: ${g}_{ii}^{\mathrm{(1)}}$, leading to the following simplified version of Eq. (1):

2 $${\rm{\Delta }}{\lambda }_{2}={e^{\prime} }_{\mathrm{2,}i}{e}_{\mathrm{2,}i}\,{g}_{ii}^{\mathrm{(1)}}={e^{\prime} }_{\mathrm{2,}i}{e}_{\mathrm{2,}i}/({k}_{i}+\mathrm{1).}$$

The smallest eigenvalue *λ*_*N*_ can be treated in a similar way. We have

3 $${\rm{\Delta }}{\lambda }_{N}={e^{\prime} }_{N,i}{e}_{N,i}{g}_{ii}^{\mathrm{(1)}}={e^{\prime} }_{N,i}{e}_{N,i}/({k}_{i}+\mathrm{1),}$$

where ***e***_*N*_ = [*e*_*N*,1_, *e*_*N*,2_, …, *e*_2,*N*_]^*T*^ is the normalized eigenvector associated with the eigenvalue ${\lambda }_{N}^{\mathrm{(0)}}$ of ***G***^(0)^: ${{\boldsymbol{G}}}^{\mathrm{(0)}}{{\boldsymbol{e}}}_{N}={\lambda }_{N}^{\mathrm{(0)}}{{\boldsymbol{e}}}_{N}$), and ${{\boldsymbol{e}}}_{N}^{-1}=[{e^{\prime} }_{N\mathrm{,1}},{e^{\prime} }_{N\mathrm{,2}},\ldots ,{e^{\prime} }_{N,N}]$ is the left eigenvector associated with ***e***_*N*_. Equations (2) and (3) characterize the impact of a single autapse on the synchronizability of the underlying neuronal network, as they show that the autapse centrality of a neuron is determined jointly by the neuron degree *k* and the two eigenvectors of the original network coupling matrix: ***e***_2_ and ***e***_*N*_.

**Figure 3:**
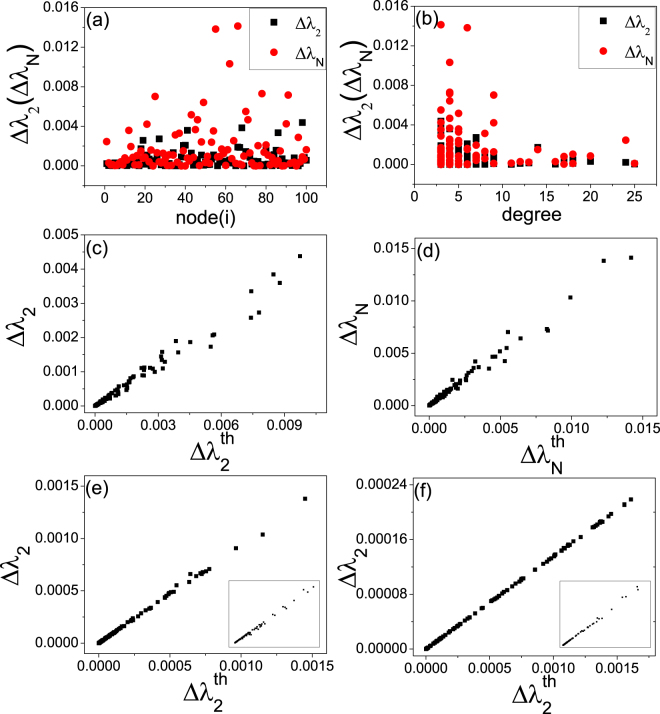
Distribution of autapse centrality for complex neuronal networks of distinct topologies. For a given network, the centrality measures can be calculated when a single autapse is attached to a node in the network. Varying the node across the network leads to a distribution of each centrality. (**a**) For a scale-free network, the distribution of Δ*λ*_2_ and Δ*λ*_*N*_, (**b**) the variations Δ*λ*_2_ and Δ*λ*_*N*_ with respect to the neuron degree *k*, (**c**) Δ*λ*_2_ with respect to the theoretical prediction Eq. (2) denoted as $\Delta {\lambda }_{2}^{th}$, and (**d**) Δ*λ*_*N*_ versus ${\rm{\Delta }}{\lambda }_{N}^{th}$, the prediction given by Eq. (3). (**e**) For an ER random neuronal network, Δ*λ*_2_ versus ${\rm{\Delta }}{\lambda }_{2}^{th}$. (**f**) For a small-world network generated by rewiring 5% of the links of a regular lattice, Δ*λ*_2_ versus ${\rm{\Delta }}{\lambda }_{2}^{th}$. The insets in (**e**) and (**f**) show Δ*λ*_*N*_ versus ${\rm{\Delta }}{\lambda }_{N}^{th}$ for the respective networks. The networks have the same size (*N* = 100) and average degree (〈*k*〉 = 6). The parameters of the neuronal dynamics are the same as those in Fig. 2.

To verify Eqs (2) and (3) numerically, we present in Fig. 3 the two autapse centrality measures for complex networks of distinct topologies. In particular, Fig. 3(a–f) are for a scale-free, random, and small-world network, respectively. The three networks have the same size *N* = 100 and average degree 〈*k*〉 = 6. Introducing a single autapse onto each neuron alternatively, we calculate the two centrality measures: the variations of the two extreme eigenvalues, Δ*λ*_2_ and Δ*λ*_*N*_. Figure 3(a,b) show the autapse centralities versus the nodal index and degree, respectively. From Fig. 3(a), we see that the centralities exhibit large variations among the neurons, while Fig. 3(b) shows that the hub neurons are less sensitive to the autapse than the non-hub neurons in spite of the large fluctuations in the values of Δ*λ*_2_ and especially of Δ*λ*_*N*_. Figure 1(c) and (d) show the numerical values of Δ*λ*_2_ and Δ*λ*_*N*_ versus the theoretical predictions of Eqs (2) and (3), respectively. For Δ*λ*_2_, the numerical and theoretical results are not exactly equal to each other but exhibit a high degree of linear correlation. For Δ*λ*_*N*_, the numerical and theoretical values are approximately equal. For the random network, the numerical results of both centralities are approximately equal to the theoretical values, as shown in Fig. 3(e). The results for the small-world network are similar to those for the scale-free network Fig. 3(f). Overall, there is a reasonable agreement between the two centrality measures calculated numerically and predicted theoretically.

**Figure 4:**
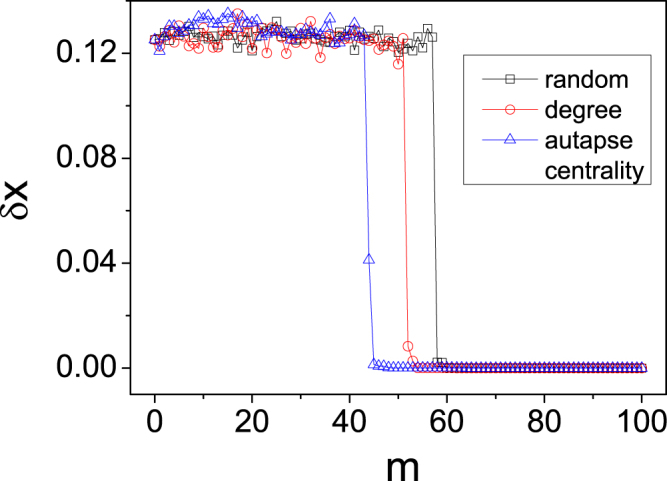
Performance of autapse centrality based strategy to optimize network synchronization. For the scale-free network in Fig. 3(a), the variation of network average synchronization error *δx* versus the number *m* of autapses for different synchronization strategies. For the autapse centrality strategy (open triangles), autapses are added to neurons in the descending order of neuron autapse centrality. For the degree (open circles) and random (open squares) strategies, the set of neurons receiving autapses is selected, respectively, according to the descending order of nodal degree and randomly. The minimum numbers of autapses for the autapse-centrality-based, degree-based, and random strategies are *m*_*c*_ = 45, 52, and 60, respectively, where the autapse centrality strategy requires the smallest number of autapses. The neuronal dynamics and coupling parameters are the same as those in Fig. 2.

We now address the issue of multiple autapses. By introducing autapses to a judiciously chosen set of neurons (one autapse to each neuron), we seek to optimize network synchronization. We again exploit Eqs (2) and (3), which suggest that the optimal set of neurons should be selected according to the largest possible values of the autapse centralities calculated based on a single autapse. Suppose the number of available autapses is *m*. We arrange the centrality values for individual nodes in a descending order and choose the *m* neurons from the top of the list. To test this optimization strategy, we compare its performance with those of two alternative strategies: degree-based and random, where for the former, the set of neurons (each receiving one autapse) are selected according to the nodal degree in the descending order, while for the latter, the set is chosen randomly. In simulations, we use the scale-free network in Fig. 3(a), with the same nodal dynamics and coupling function as in Fig. 2(a). As the stable region of the MSF curve is bounded Fig. 2(a), the relevant autapse centrality is Δ*λ*_*N*_: the set of neurons to the receive the autapses can be chosen according to the descending order of the values of Δ*λ*_*N*_. We calculate the network-averaged synchronization error of the variable *x*: $\langle \delta x\rangle \equiv {\langle \mathrm{(1/}N){\sum }_{i\mathrm{=1}}^{N}|{x}_{i}(t)-{x}_{ave}(t)|\rangle }_{T}$, with *x*_*ave*_(*t*) being the nodal average value and 〈...〉_*T*_ being the time average. Our computations reveal that, as the number of autapses *m* is increased through a critical point (denoted as *m*_*c*_), the synchronization error decreases to zero, as shown in Fig. 4. For our autapse centrality based optimization strategy, we have *m*_*c*_ ≈ 45, while the degree based and random strategies give *m*_*c*_ ≈ 52 and *m*_*c*_ ≈ 60, respectively. Among the three strategies, ours yields the minimum number of autapses required for the network to be fully synchronized.

### Generality and robustness of autapse-centrality based synchronization strategy

We address a number of issues to further substantiate the main result that autapses could promote global synchronization in neuronal networks, including: (1) the performance of autapse-centrality based synchronization strategy for other types of complex networks; (2) the impacts of parameter mismatches among neurons on global synchronization; and (3) the influences of the system parameters on the minimum number of autapses required for global synchronization.

**Figure 5:**
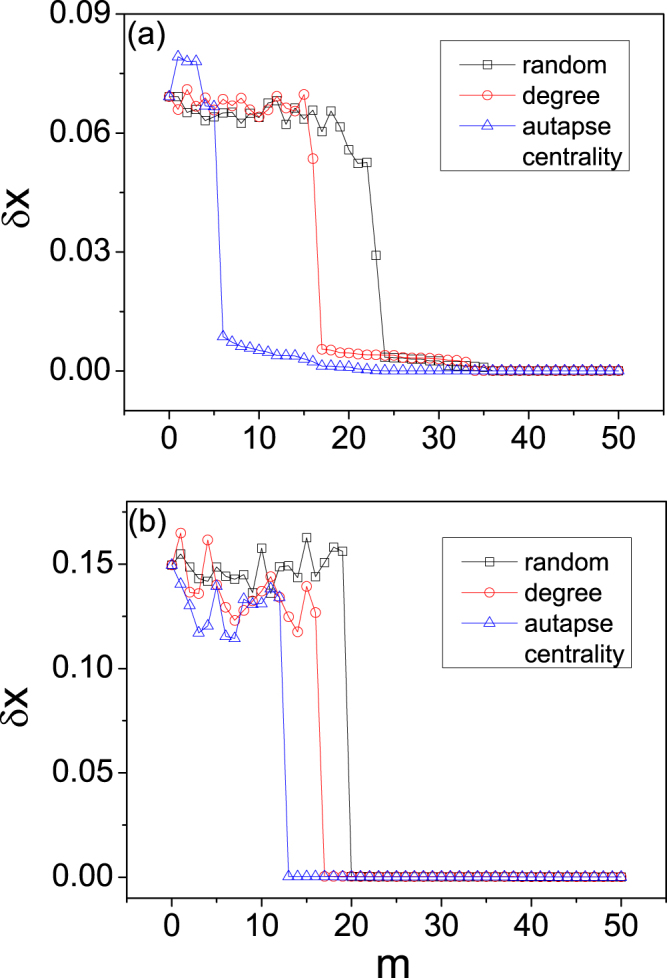
Performance of autapse-centrality based synchronization strategy for the random and small-world networks. The size, average degree, local dynamics and coupling function of the networks are identical to those for scale-free networks Fig. 4. (**a**) Results for random networks. The parameters are: (*ε*, *τ*) = (0.95, 4). The minimum numbers of autapses for the autapse-centrality based, degree based and random strategies are *m*_*c*_ = 23, 34 and 36, respectively. (**b**) Results for small-world network, which is constructed with the rewiring probability *p* = 0.1 for (*ε*, *τ*) = (0.95, 1.8). The minimum numbers of autapses for the autapse-centrality based, degree based and random strategies are *m*_*c*_ = 13, 17 and 20, respectively. For both the random and small-world networks, the autapse-centrality based strategy requires the least number of autapses in achieving global synchronization.

So far we have demonstrated that the autapse-centrality based strategy outperforms the conventional degree based and random strategies in promoting global synchronization in scale-free network, as shown in Fig. 4. Our theoretical analysis implies that this result should hold for the general network model, regardless of the network topologies. An example is given in Fig. 5, which displays the synchronization performance of the three strategies for the random and small-world networks. We see that, as for the case of scale-free networks in Fig. 4, the autapse-centrality based strategy always requires the least number autapses in achieving global network synchronization.

**Figure 6:**
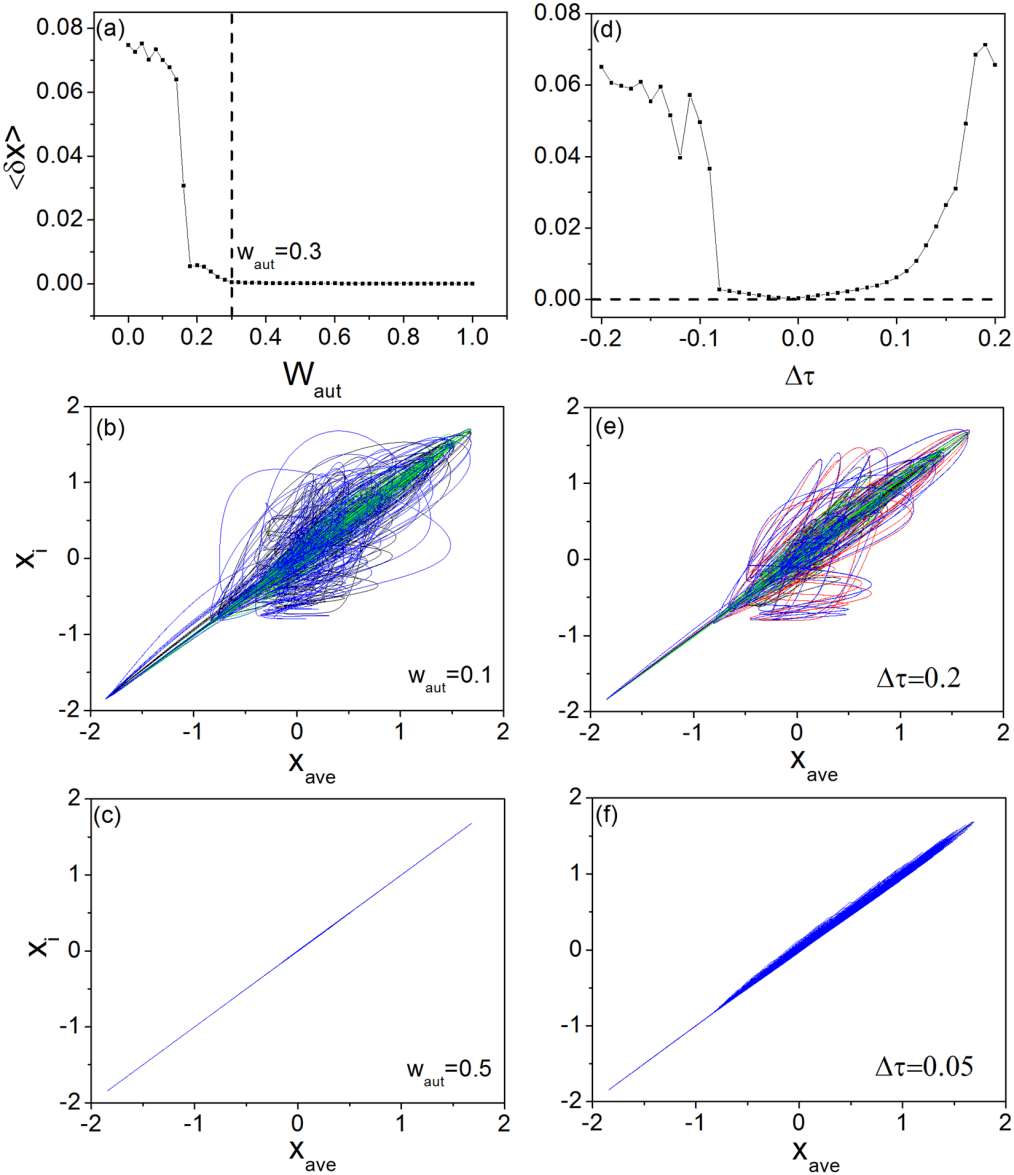
Impacts of parameter mismatch on synchronization. Two types of parameter mismatch are considered for the toy network in Fig. 1: in the coupling strength and in the time delay. The values of these two parameters associated with the synaptic connections are fixed at (*w*, *τ*) = (1, 4), whereas those of the autaptic connection, denoted as *w*_*aut*_ and *τ*_*aut*_, respectively, are varied. (**a**) For fixed *τ*_*aut*_ = 4, the network synchronization error 〈*δx*〉 versus *w*_*aut*_, where 〈*δx*〉 = 0 for *w*_*aut*_ > 0.3. (**b**) For *w*_*aut*_ = 0.1, the neurons are desynchronized. (**c**) For *w*_*aut*_ = 0.5, the neurons are completely synchronized. (**d**) For fixed *w*_*aut*_ = 1, 〈*δx*〉 versus Δ*τ* = *τ*_*aut*_ − *τ*_*aut*_. The neurons are desynchronized when Δ*τ* is large (**e**) but they are well synchronized when Δ*τ* is small (**f**).

In real systems, parameter mismatches among the neuronal dynamics and the network links are inevitable. To gain insights into whether autapses could promote synchronization in networks consisting of non-identical neurons, we consider the toy network model in Fig. 1 and introduce small mismatch in the parameters (*I*, *ε*, and *τ*). To be concrete, we vary the coupling strength *w*_*aut*_ and the time delay *τ*_*aut*_ of the autapse and investigate how network synchronization is deteriorated as the values of *w*_*aut*_ and *τ*_*aut*_ are deviated from those of the synaptic connections. Setting *w* = 1 and *τ* = 4 for the synaptic connections, we calculate the synchronization error 〈*δx*〉 as a function of *w*_*aut*_. The numerical results are shown in Fig. 6(a). We observe 〈*δx*〉 = 0 for *w*_*aut*_ > 0.3. To see how mismatched coupling strength affects synchronization, we plot in Fig. 6(b) and (c) the neuron trajectories for *w*_*aut*_ = 0.1 and *w*_*aut*_ = 0.5, respectively, where there is still global synchronization for the latter but for the former, synchronization is lost. This indicates that, even when the mismatch of *w*_*aut*_ is about 50% Fig. 6(c), synchronization can still be achieved. In fact, given *w*_*aut*_ > 0.3, global synchronization will be maintained regardless of the mismatch amplitude Fig. 6(a). The underlying reason for the robustness of global synchronization to the mismatch of *w*_*aut*_ is that, due to the normalized coupling scheme (see Methods), the dynamics of the synchronous manifold is independent of the autapse strength.

Figure 6(d) demonstrates the robustness of network synchronization in the presence of mismatch in time delay, where the behavior of 〈*δx*〉 versus Δ*τ* = *τ*_*aut*_ − *τ*_*aut*_ is shown for fixed *w*_*aut*_ = 1. We see that the network is well synchronized for a small amount of mismatch in the time delay. For example, we have 〈*δx*〉 < 0.1 for Δ*τ* ∈ (−0.08, 0.1). For a relatively large amount of mismatch, synchronization is lost, as shown in Fig. 6(e) for Δ*τ* = 0.2. In general, for Δ*τ* ≠ 0, the local dynamics of the autapsed neuron are different from those of the other neurons, making global synchronization difficult. However, as shown in Fig. 6(f), if the mismatch amount Δ*τ* is not too large, all neurons in the network can still be well synchronized.

**Figure 7:**
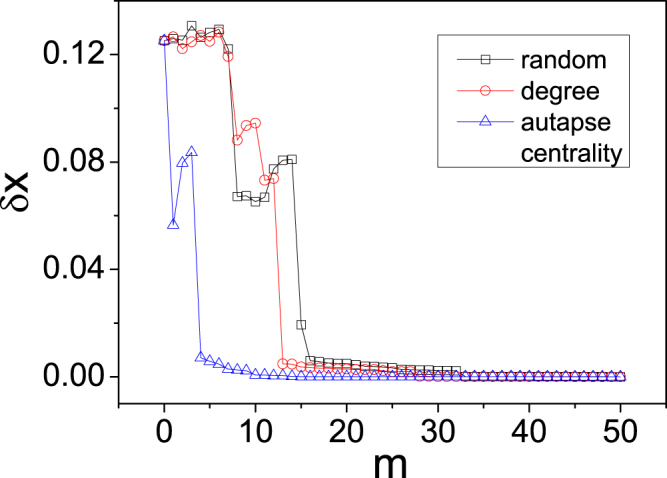
Example of how the change of network parameter could reduce the critical number of autapses required for global synchronization. For the same network structure and the value of the parameter *τ* used in Fig. 4 but with a change in the coupling strength to *ε* = 0.95, the network averaged synchronization error *δx* versus the number of autapses for the three synchronization strategies. For the autapse-centrality based, degree based, and random strategies, the critical numbers of autapses are *m*_*c*_ = 19, 31, and 35, respectively, signifying a marked reduction in comparison with the results in Fig. 4.

In general, the number of autapses required to synchronize a complex network depends on the network parameters such as the connecting density, the degree distribution, the delay parameter, and the coupling strength among the neurons. For the scale-free network in Fig. 4 of 100 nodes and average degree 〈*k*〉 = 6 and by the parameters *ε* = 1 and *τ* = 4, *m*_*c*_ = 45 autapses are required for global synchronization. This critical number of autapses, however, could be changed significantly by varying the network parameters. To shown an example, we fix the network structure and the parameter *τ* used in Fig. 4 but changing the value of *ε* to 0.95, and recalculate the number of required autapses for different synchronization strategies. The numerical results are shown in Fig. 7. We see that, for the autapse-centrality based, degree based, and random strategies, the critical numbers of autapses required for global synchronization are reduced to, respectively, to *m*_*c*_ = 19, 31, and 35.

## Discussion

In this paper, we study neuronal autapses, a type of structural anomalies that give certain neurons a time-delayed, self-feedback loop. Autapses, when they were first discovered^1^, were thought of playing no specific biological or physiological role. Only later were their potential impacts in biological functions found or suggested^3–8^. In view of the fundamental importance of synchrony in biology, physiology, and biomedical sciences, we focus our study on the possible role of autapses in modulating neuronal synchrony through computation and theory. Our main finding is that, for a complex neuronal network, even the existence of autapses on a small fraction of the neurons can promote synchrony significantly. In particular, using both a small toy network and larger networks of distinct complex topologies, we introduce autapse to a fraction of neurons and analyze the change in the network synchronizability. Based on a systematic analysis of the impact of a single autapse at different nodes in the network, we develop centrality measures to analyze the situation of multiple autapses in terms of their optimal placement in the network to realize robust synchronization of the entire network. Our work provides the computational and theoretical foundation for hypothesizing the positive role of autapses in promoting synchronization in neuronal networks.

It is an accepted notion that synchrony in neuronal networks is strongly correlated with certain neurological diseases such as epileptic seizures^18^, but whether such a disease can be attributed to an increasing level of synchrony has been a controversial issue^19–22^. At the present it remains difficult to ascertain, through the traditional approach of EEG or ECoG data analysis, whether a general, well defined causal relation between an elevation of synchrony and the occurrence of seizure exists. The main idea underlying our work is to focus on “unusual” features or structural anomalies in neurons and to study their role in modulating neuronal synchrony. Suppose for certain type of seizure, the responsible neuronal network in a brain region can be identified. Whether there exist structural anomalies in some neurons in this region can then lead to insights into the interplay between synchrony and the particular type of seizure. Generally, the issue of synchronization goes much beyond the scope of epileptic seizures with broad implications to biology and physiology^16^.

We discuss a few technical issues. Firstly, to make the model theoretically tractable, we assume that the isolated neuronal dynamics are identical and the links (including the autapses) have the same coupling strength and time delay. However, in real systems, parameter mismatches among the neuronal dynamics and network links can be expected. We have gained preliminary insights into whether autapses can promote synchronization in networks of non-identical neurons, by introducing small mismatches in parameters (*I*, *ε*, and *τ*) in the toy network model in Fig. 1. Our computations reveal, for example, when the time delay parameter has a 2% mismatch among the network links, global synchronization can still be achieved. Secondly, in our study we adopt the scheme of normalized couplings to enable computation and analysis of the MSF and the proposal of autapse centralities. If the couplings are not normalized, such a theoretical analysis would not be possible and it appears at the present that one must rely on numerical simulations to uncover and quantify the role of autapses in synchronization. Thirdly, we assume linear feedback couplings in the network. While this type of coupling scheme does have physiological support (e.g., in terms of electrical synapses underlying the neuronal connections), it remains unknown whether other types of couplings, e.g., those with chemical synapses, can lead to effective modulation of network synchronization by autapses.

Finally, we point out the significance of our work in general network science and engineering with respect to the problems of synchronization and control. A focus in this area of research has been on the role of long-range connections, such as those in small-world networks^53^, in promoting network synchronization^36^. From a practical standpoint, long range connections may be costly^54^. For example, for neural networks in the human brain^55^, while there are white fiber tracts among distant cortical areas which are essential for the brain functions, the network connectivity is still dominated by short-distance, local connections, due to the minimization principle of the axonal length and energy consumption. A similar issue arises in other realistic systems^54^ such as traffic networks, power grids, communication network, and the Internet. Our finding that autapses, the shortest possible links in any network, can play a positive role in promoting synchronization, is encouraging from the perspective of optimal control of complex network dynamics at a minimal cost.

## Methods

### Model description

Our model of neuronal network with autapses reads

4 $${\dot{{\boldsymbol{x}}}}_{i}={{\boldsymbol{F}}}_{i}({{\boldsymbol{x}}}_{i})+\frac{\varepsilon }{{k}_{i}+{\delta }_{il}}\sum _{j\mathrm{=1}}^{N}{c}_{ij}[{\boldsymbol{H}}({{\boldsymbol{x}}}_{j})(t-{\tau }_{ij})-{\boldsymbol{H}}({{\boldsymbol{x}}}_{i})]+{\eta }_{i}(t),$$

where *i*, *j* = 1, 2, …, *N* are nodal indices, *N* is the network size, ***x***_*i*_ the state vector of the *i* th neuron, *ε* is the coupling parameter, ***F***_*i*_(***x***) is the velocity field governing the dynamics of the *i* th neuron when it is isolated, ***H***(***x***) is the coupling function, *τ*_*ij*_ denotes the time delay of signal propagating from neuron *j* to *i*, and *η*_*i*_(*t*) represents the independent, identically distributed Gaussian white noise added to the membrane potential of each neuron, with 〈*η*_*i*_(*t*)〉 = 0 and 〈*η*_*i*_(*t*)*η*_*j*_(*t*)〉 = (2 × 10^−10^)*δ*_*ij*_. The network structure in the presence of autaptic connections is characterized by the coupling matrix ***C*** = {*c*_*ij*_}. For non-diagonal elements, we have *c*_*ij*_ = *c*_*ji*_ = 1 if there is a link between neurons *i* and *j*, otherwise *c*_*ij*_ = 0. For the diagonal elements, we set *c*_*ii*_ = *δ*_*il*_, where *δ*_*il*_ is the Kronecker delta function and ***V*** = {*l*} (with *l* = 1, …, *m*) denotes the set of neurons with autaptic connections. Specifically, we have *c*_*ii*_ = 1 if neuron *i* possesses an autapse, and *c*_*ii*_ = 0 otherwise. The quantity *k*_*i*_ = ∑_*j* ≠ *i*_*c*_*ij*_ is the number of links connected to neuron *i* (i.e., the degree). To capture the essential network dynamics subject to autapses while making the model theoretically tractable, we assume identical time delays: *τ*_*ij*_ = *τ*. Biologically, this approximation is reasonable for synapses within the same cortical minicolumn^55^. The scheme of diffusive (linear feedback) coupling assumed in Eq. (4) models the realistic electrical synaptic interactions (e.g., of the gap-junction type) among neurons^16,56^.

In simulations, we set the parameters of the HR oscillator as (*a*, *b*, *c*, *d*, *r*, *s*, *x*_*R*_, *I*) = (1, 3, 1, 5, 6 × 10^−3^, −1.6, 3.2), for which the isolated HR neuron exhibits chaotic bursting dynamics^28^. The coupling function is chosen to be ***H***(***x***) = [*x*, 0, 0]^*T*^, i.e., the neurons in the network are coupled through their membrane potentials. We use the Bogacki-Shampine algorithm^57^ to simulate Eq. (4), which is a time-delayed, stochastic system of coupled differential equations. The integration time step is *δt* = 1 × 10^−3^.

### Persistence of a global synchronization manifold in the presence of an autapse

We argue that, given that the autapses have an identical time delay with respect to the synaptic connections, the neuronal network possesses a synchronization manifold. Consider the network in Fig. 1(b1), in which the dynamics of neuron 1 are governed by the equation

$${\dot{{\boldsymbol{x}}}}_{1}=\{{{\boldsymbol{F}}}_{1}({{\boldsymbol{x}}}_{1})+\frac{\varepsilon }{4}[{x}_{1}(t-\tau )-{x}_{1}]\}+\frac{\varepsilon }{4}\sum _{j\ne 1}[{x}_{j}(t-\tau )-{x}_{1}],$$

where the 1st and 2nd terms on the right side represent, respectively, the local dynamics of the autapsed neuron and the coupling signals it received from the neighboring neurons. Assume that the network is globally synchronized, and denote ***x***_*s*_ as the dynamical state within the synchronous manifold, we have ***x***_*i*_(*t* − *τ*) = ***x***_*s*_(*t* − *τ*) (for *i* = 1, …, 4). Equation (5) can then be rewritten as

$${\dot{{\boldsymbol{x}}}}_{1}={{\boldsymbol{F}}}_{1}({{\boldsymbol{x}}}_{1})+\frac{\varepsilon }{4}[{x}_{s}(t-\tau )-{x}_{1}]+\frac{\varepsilon }{4}\sum _{j\ne 1}[{x}_{s}(t-\tau )-{x}_{1}]$$

5 $$={{\boldsymbol{F}}}_{1}({{\boldsymbol{x}}}_{1})+\frac{\varepsilon }{4}\sum _{j\mathrm{=1}}^{4}[{x}_{s}(t-\tau )-{x}_{1}]={{\boldsymbol{F}}}_{1}({{\boldsymbol{x}}}_{1})+\varepsilon [{x}_{s}(t-\tau )-{x}_{1}\mathrm{].}$$

For neurons without autapse (*j* = 2, 3, 4), their dynamics are governed by

6 $${\dot{{\boldsymbol{x}}}}_{j}={{\boldsymbol{F}}}_{j}({{\boldsymbol{x}}}_{j})+\frac{\varepsilon }{{k}_{j}}\sum _{l\mathrm{=1}}^{4}{c}_{jl}[{x}_{s}(t-\tau )-{x}_{j}]={{\boldsymbol{F}}}_{j}({{\boldsymbol{x}}}_{j})+\varepsilon [{x}_{s}(t-\tau )-{x}_{j}],$$

which is identical to Eq. (5). That is, in the synchronous state all neurons in the network, regardless of whether they are autapsed or not, follow the same dynamical equation. Further, because of the normalized coupling scheme, the equations are independent of the network structure. A global synchronization manifold thus persists in the presence of autapses, regardless of their locations in the network.

### Master stability function based analysis of network synchronization in the presence of autapses

To uncover the impact of autapses on network synchronization quantitatively, we analyze the stability of the synchronous manifold. Within the manifold, the dynamics are governed by

7 $${\dot{{\boldsymbol{x}}}}_{s}={\boldsymbol{F}}({{\boldsymbol{x}}}_{s})+\varepsilon [{\boldsymbol{H}}({{\boldsymbol{x}}}_{s}(t-\tau ))-{\boldsymbol{H}}({{\bf{x}}}_{s}\mathrm{)].}$$

Let *δ****x***_*i*_ = ***x***_*i*_ − ***x***_*s*_ be an infinitesimal perturbation to ***x***_*s*_, whose evolution is governed by the variational equations obtained by linearizing Eq. (4) about ***x***_*s*_:

8 $$\delta {\dot{{\boldsymbol{x}}}}_{i}=[{\boldsymbol{DF}}({{\boldsymbol{x}}}_{s})-\varepsilon {\boldsymbol{DH}}({{\boldsymbol{x}}}_{s})]\delta {{\boldsymbol{x}}}_{i}+\varepsilon \sum _{j\mathrm{=1}}^{N}{g}_{ij}{\boldsymbol{DH}}({{\boldsymbol{x}}}_{s}(t-\tau ))\delta {{\boldsymbol{x}}}_{j}(t-\tau ),$$

for *i*, *j* = 1, …, *N*, where ***G*** = {*g*_*ij*_} = {*c*_*ij*_/(*k*_*i*_ + *δ*_*il*_)} is the normalized coupling matrix, ***DF*** and ***DH*** are the Jacobians of the isolated neuron dynamics and of the coupling function, respectively. Projecting {*δ****x***_*i*_} into the eigenspace spanned by the eigenvectors of ***G***, we obtain *N* independent variational equations

9 $$\delta {\dot{{\boldsymbol{y}}}}_{i}=[{\boldsymbol{DF}}({{\boldsymbol{x}}}_{s})-\varepsilon {\boldsymbol{H}}({{\boldsymbol{x}}}_{s})]\delta {{\boldsymbol{y}}}_{i}+\varepsilon {\lambda }_{i}{\boldsymbol{DH}}({{\boldsymbol{x}}}_{s}(t-\tau ))\delta {{\boldsymbol{y}}}_{i}(t-\tau ),$$

where *δ****y***_*i*_ is the *i* th mode of the perturbation, and 1 = *λ*_1_ > *λ*_2_ ≥ ... ≥ *λ*_*N*_ are the eigenvalues of ***G***. The mode associated with *λ*_1_ describes the chaotic motion within the synchronous manifold as corresponding to the dynamics of each isolated neuron, and the transverse modes {*λ*_*j*_} (*j* = 2, …, *N*) determine the stability of the synchronous chaotic motion within the synchronization manifold. Denote Λ_*j*_ as the largest Lyapunov exponent calculated from the variational equations for the *j* th mode: the mode is stable (unstable) if Λ_*j*_ < 0 (Λ_*j*_ > 0). The necessary condition for the synchronous state to be stable is that all the transverse modes decay exponentially with time: Λ_*j*_ < 0 for *j* = 2, …, *N*. Note that Λ is determined by three parameters (*ε*, *τ*, and *λ*), which is different from systems without any autapse^49,50,58^ in which Λ depends only on the generic coupling strength *σ* = *ελ*. For the autapsed network, we determine its synchronizability by two steps: (1) finding the stable region of the MSF curve in the parameter space (*ε*, *λ*, *τ*) and (2) calculating the eigenvalues {*λ*_*i*_} of the normalized coupling matrix ***G***, where the former depends on both the local dynamics and the coupling function but the latter is solely determined by the network structure.
